# Causal relationship between hydrogen sulfide and osteoarthritis: A Mendelian randomization analysis

**DOI:** 10.1097/MD.0000000000047626

**Published:** 2026-02-13

**Authors:** Peng Yang, Ye Jiang, Jun Ma, Xiaofei Ye, Lina Liu, Jiuyi Sun

**Affiliations:** a Department of Orthopaedics, Naval Medical Center of PLA, Naval Military Medical University, Shanghai, China; b Department of Health Statistics, Naval Medical University, Shanghai, China.

**Keywords:** gut bacterial pathway abundance, hydrogen sulfide, Mendelian randomization, osteoarthritis

## Abstract

Hydrogen sulfide (H_2_S) has been shown to alleviate bone and joint inflammation in osteoarthritis (OA). Exploring the connection between H_2_S and OA could reveal novel therapeutic avenues for treating this condition. This study utilized H_2_S as the exposure variable and OA as the outcome within a Mendelian randomization framework. Data for genome-wide association studies related to both exposure and outcome were sourced from the IEU OpenGWAS project. To estimate causal relationships, inverse variance weighted method was employed, along with supplementary analytical techniques (MR-Egger, weighted median, simple mode, and weighted mode), and sensitivity studies were carried out to evaluate our results’ dependability. The inverse variance weighted analysis revealed that the abundance of a specific gut bacterial pathway involved in the reduction process of sulfate “SO4ASSIM.PWY..sulfate.reduction.I..assimilatory.” acts as a protective factor against hospital-diagnosed knee osteoarthritis, with an odds ratio of 0.687 (95% confidence interval = 0.479–0.987, *P* = .042). In contrast, gut bacterial pathway abundance associated with sulfate assimilation and cysteine biosynthesis “SULFATE.CYS.PWY..superpathway.of.sulfate.assimilation.and.cysteine.biosynthesis” was linked to increased risk for hospital-diagnosed OA, showing an odds ratio value of 1.120 (95% confidence interval = 1.000–1.255, *P* = .049). Sensitivity analyses validated the strength of the causal relationship between the gut bacterial pathway abundance and OA. We identified that the gut bacterial pathway abundance associated with H_2_S “SO4ASSIM.PWY..sulfate.reduction.I..assimilatory.” acts as a protective factor for OA suggesting its potential role in OA treatment and offering new avenues for research on H_2_S in the treatment of OA in this context.

## 1. Introduction

Osteoarthritis (OA) is a prevalent musculoskeletal disorder predominantly affecting the elderly and ranking among the leading causes of joint disability.^[1]^ The key pathological alterations in OA encompass cartilage degeneration accompanied by subchondral bone sclerosis and synovitis. Globally, over 300 million individuals are estimated to suffering from OA, with the annual cost of treatment exceeding 460 billion US dollars, thereby imposing a substantial economic burden on both families and society.^[2,3]^ Despite its multifactorial etiology, which encompasses metabolic, genetic, epigenetic, and local environmental factors, the precise pathogenic mechanisms of OA to be fully elucidated. Presently available therapeutic strategies for OA include physical exercise and pharmacological interventions; however, advanced-stage patients often require joint replacement surgeries.^[4]^ This highlights the urgent necessity for conducting comprehensive research on the pathogenesis and regulatory mechanisms of OA to establish a scientific basis for implementing more targeted intervention strategies.

Hydrogen sulfide (H_2_S), a crucial gaseous signaling molecule, exerts significant roles in a wide array of physiological and pathological processes.^[5,6]^ Endogenously, H_2_S is synthesized by several essential enzymes, namely cystathionine b-synthase, cystathionine c-lyase, and 3-mercaptopyruvate sulfurtransferase.^[7]^ Endogenous H_2_S is a new inflammatory mediator and has been found to play an important role in a variety of inflammatory diseases, including joint inflammation/arthritis.^[7]^ Studies have shown that high concentrations of H_2_S (above 0.5 mM) exacerbate OA inflammation by activating the MAPK/NF-kB pathway.^[8,9]^ In addition, H_2_S is also crucial in modulating bone tissue function, maintaining bone anabolism and homeostasis through the epigenetic differentiation of bone marrow mesenchymal stem cells.^[10]^ Several studies have demonstrated that the endogenous H_2_S levels in OA bone tissue are diminished, and H_2_S supplementation can alleviate inflammation, oxidative stress, and pain associated with OA progression, which accentuates the involvement of H_2_S in the development of OA and emphasizes its important pathophysiological significance.^[11–13]^ Interestingly, some studies have shown that the plasma H_2_S levels in OA patients are significantly higher than in healthy controls,^[14]^ while Burguera et al^[5]^ suggest that the biosynthesis of H_2_S may be reduced in OA joints. It has been hypothesized that an increase in H_2_S in inflammatory diseases may represent an endogenous compensatory mechanism by which cells attempt to counteract inflammation. Given that the causal relationship between H_2_S and OA remains unclear, conducting Mendelian randomization (MR) study to clarify the direct relationship between them and provide stronger evidence is crucial for understanding the role of H_2_S in OA.

MR is an innovative analytical method that can provide an unbiased estimation of the causal relationship between phenotypes.^[15]^ As a genetic epidemiology technique, MR utilizes single nucleotide polymorphisms (SNPs) that are highly correlated with exposure as instrumental variables (IVs) to infer the causal impact of exposure on the outcome. This study employs MR analysis to determine whether there is a causal relationship between H_2_S and the occurrence of OA, providing further insights into the role of H_2_S in OA.

## 2. Materials and methods

### 2.1. Data source

Data from GWAS concerning H_2_S and OA were retrieved from the IEU OpenGWAS project website (https://gwas.mrcieu.ac.uk/). Data of exposure factors associated with H_2_S comprised 3 datasets (met-a-455, prot-a-1933, and ebi-a-GCST90027644), with sample sizes ranging from 3301 to 7738 European individuals. OA data included 8 datasets (ebi-a-GCST90013881, ebi-a-GCST90013931, ebi-a-GCST90038686, ebi-a-GCST007091, ebi-a-GCST007090, ebi-a-GCST005814, ebi-a-GCST005813, and ebi-a-GCST005810), encompassing 11,989 to 484,598 European individuals. Detailed information was provided in Table 1. This study relies on publicly available GWAS summary statistics as the data source. Since no new data were collected for this research, no additional ethical approval was required.

**Table 1:** Genome-wide association studies (GWAS) data information for hydrogen sulfide (H_2_S) and osteoarthritis (OA).

Traits	GWIS_ID	ncase	ncontrol	Number of SNPs
Cysteine	met-a-455	7692		2,545,727
3-mercaptopyruvate sulfurtransferase	prot-a-1933	3301		10,534,735
Gut bacterial pathway abundance (SO4ASSIM.PWY..sulfate.reduction.I..assimilatory.)	ebi-a-GCST90027643	7738		5,563,593
Gut bacterial pathway abundance (SULFATE.CYS.PWY..superpathway.of.sulfate.assimilation.and.cysteine.biosynthesis)	ebi-a-GCST90027644	7738		5,563,685
Osteoarthritis (Firth correction)	ebi-a-GCST90013881	407,746		11,039,204
Osteoarthritis (SPA correction)	ebi-a-GCST90013931	407,746		11,039,197
Osteoarthritis	ebi-a-GCST90038686	39,515	445,083	9,587,836
Osteoarthritis (hip)	ebi-a-GCST007091	15,704	378,169	29,771,219
Knee osteoarthritis	ebi-a-GCST007090	24,955	378,169	29,999,696
Osteoarthritis (hospital diagnosed)	ebi-a-GCST005814	10,083	40,425	15,845,511
Osteoarthritis of the knee (hospital diagnosed)	ebi-a-GCST005813	4462	17,885	15,708,690
Osteoarthritis of the hip (hospital diagnosed)	ebi-a-GCST005810	2396	9593	15,543,682

ncase = number of diseased samples, ncontrol = number of control samples, SNP = single nucleotide polymorphism.

### 2.2. IVs selection

In this study, H_2_S was designated as the exposure variable, while OA was considered as the outcome variable for MR analysis. This study was developed based on 3 key assumptions: a significant correlation exists between IVs and H_2_S; IVs are independent of potential confounders; and IVs only affect OA through H_2_S. Strict thresholds can over-screen affecting the statistical power of subsequent analyses, so we screened SNPs with *P*-values < 1 × 10^−5^ that showed a significant correlation with H_2_S.^[16,17]^ Subsequently, the ld_clump() function from the ieugwasr package (v 0.1.5)^[18]^ was employed to eliminate SNPs with linkage disequilibrium, with parameters set at *r*^2^ = 0.001 and kb = 10,000. Finally, the *F*-value was used to evaluate IV strength, discarding weak IVs with *F*-statistics < 10. Potential confounders related to the outcome were filtered using data from the GWAS Catalog database (https://www.ebi.ac.uk/gwas/), employing a *P*-value < 1 × 10^−5^ as a threshold. Additionally, the Steiger test was applied to confirm the directionality of the causal relationship. We adjusted non-palindromic IV chains and eliminated palindromic IVs to ensure consistent allele correspondence and order for exposure and outcome.

### 2.3. Statistical analysis

**Figure 1.**
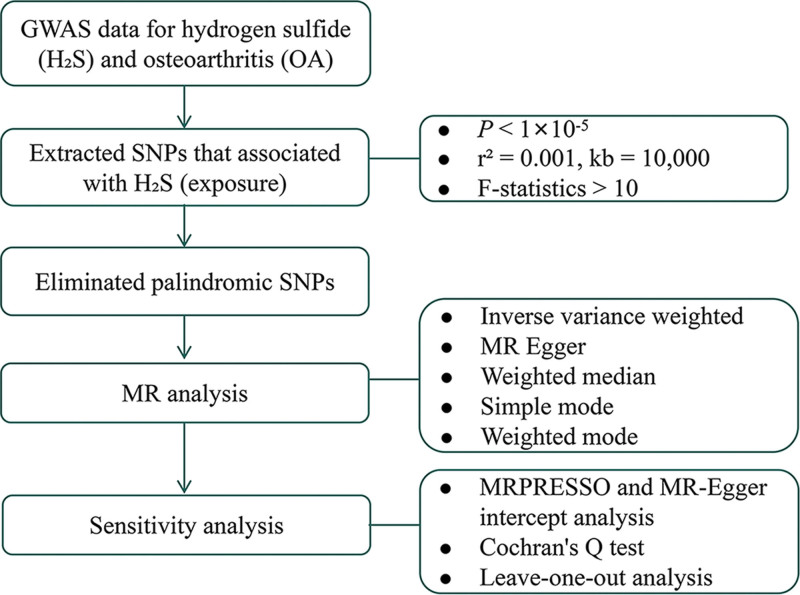
Study design and workflow. MR = Mendelian randomization, SNP = single nucleotide polymorphism

Potential causal effects were evaluated employing 5 distinct methodologies: inverse variance weighted,^[19]^ MR-Egger,^[20]^ weighted median,^[21]^ simple mode,^[22]^ and weighted mode.^[22]^ MR-PRESSO was used to detect and remove outliers to mitigate horizontal pleiotropy in subsequent analyses. Sensitivity analyses were performed to mitigate potential sources of bias. The mr_heterogeneity() function assessed heterogeneity, applying random effects when *P*-value < .05, and fixed effects otherwise. Horizontal pleiotropy was tested using MR-Egger regression intercept, with *P*-values < .05 indicating pleiotropy. A leave-one-out method was employed for recalculating results after systematically removing individual SNPs. Causal effects were quantified using odds ratios (OR) accompanied by 95% confidence intervals (CIs). The flowchart of the study is presented in Figure 1. The statistical analyses were predominantly conducted using the TwoSampleMR package (v 0.6.0)^[23]^ and the MR-PRESSO package (v 1.0).^[24]^

## 3. Results

### 3.1. Characteristics of selected IVs

MR was employed to assess the causal relationship between H_2_S and OA, identifying 504 SNPs as IVs for further analysis, with SNP utilization ranging from 32 to 187. F-statistics ranged from 19.072 to 32.072, confirming strong associations between selected IVs and the exposure, as shown in Tables S1 and S2, Supplemental Digital Content, https://links.lww.com/MD/R360.

### 3.2. *Causal effect of exposure factors associated with H*_*2*_*S on OA*

**Figure 2.**
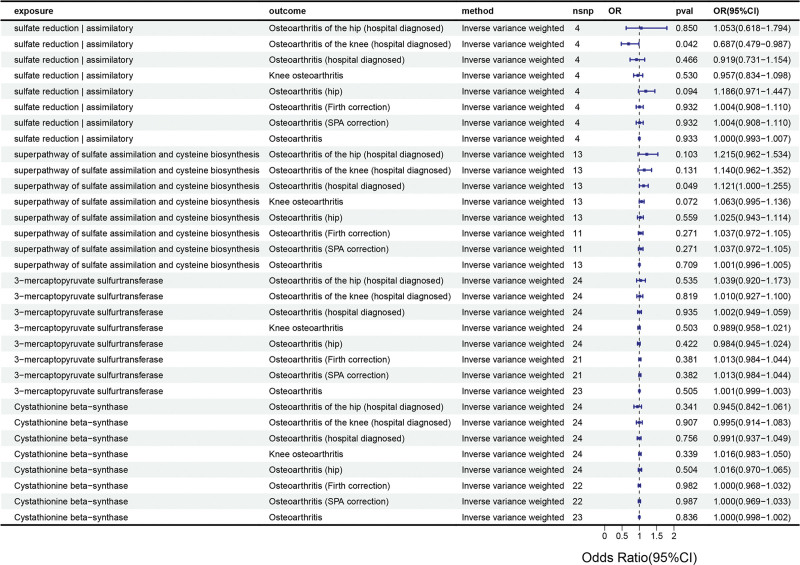
Forest plot of MR results. MR = Mendelian randomization, SNP = single nucleotide polymorphism, OR = odds ratio, CI = confidence interval, *P*val = significance *P*-value.

MR analysis revealed 2 significant causal relationships between H_2_S-related exposure factors and OA. Inverse variance weighted results indicated that the gut bacterial pathway abundance “SO4ASSIM.PWY..sulfate.reduction.I..assimilatory.” was a protective factor for knee osteoarthritis (KOA; hospital diagnosed; OR = 0.687, 95% CI = 0.479–0.987, *P* = .042), while another gut bacterial pathway “SULFATE.CYS.PWY..superpathway.of.sulfate.assimilation.and.cysteine.biosynthesis” abundance was identified as a risk factor for OA (hospital diagnosed; OR = 1.120, 95% CI = 1.000–1.255, *P* = .049; Fig. 2).

### 3.3. Robustness of MR analysis results

**Figure 3.**
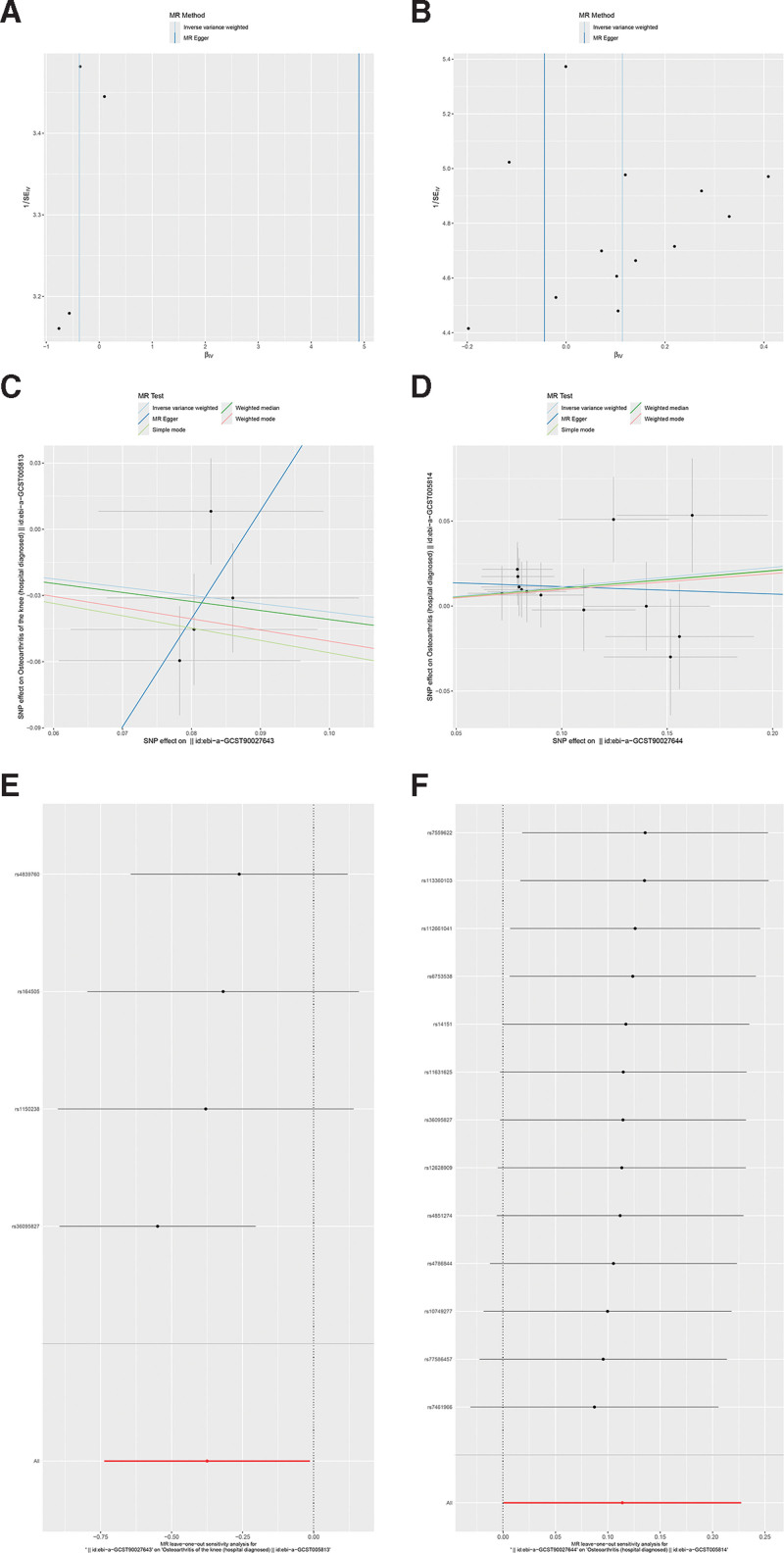
The results of funnel plots, scatter plots, and leave-one-out tests in MR analysis. (A and B) Funnel plots for visualizing heterogeneity of instrumental variables. (C and D) Scatter plots of MR results. The slope of each line signifies the causality of the respective method. (E and F) Leave-one-out analysis for exposure on outcome. Each row in the figure is meant to be the effect of the model when that row of SNPs is removed. MR = Mendelian randomization, SNP = single nucleotide polymorphism

To validate the robustness of our findings, we employed MR-Egger intercept analyses for potential multiple influences, which revealed no evidence of pleiotropy (*P* > .05). Additionally, the Cochran *Q* test indicated no significant heterogeneity (*P* > .05), and IVs exhibited symmetry in the funnel plots (Fig. 3A and B). Scatter plots illustrated the directional analysis of the 5 MR algorithms (Fig. 3C and D). Leave-one-out analysis, which progressively removed individual SNPs, showed minimal effect on the outcome variable (Fig. 3E and F), reinforcing the robustness and credibility of our study results.

## 4. Discussion

OA is a degenerative joint disorder that severely restricts the daily activities of the elderly. Studies have shown that patients with knee or hip OA exhibit a higher all-cause mortality rate compared with the general population.^[1,3]^ Unfortunately, there is currently no radical cure for OA. A substantial body of evidence has indicated a close association between endogenous H_2_S and OA development.^[5,25,26]^ H_2_S, acting as a gaseous transmitter, plays a role in modulating inflammation, oxidative stress, and pain related to OA.^[27,28]^ It is now recognized that at different concentrations, H_2_S exerts both anti- and pro-inflammatory effects.^[7]^ In light of this, we conducted an MR analysis to explore the potential causal relationship between H_2_S-related exposure factors and OA. Our main finding revealed that gut bacterial pathway abundance “SO4ASSIM.PWY..sulfate.reduction.I..assimilatory.” served as a protective factor for KOA. Conversely, another gut bacterial pathway abundance associated with sulfate assimilation and cysteine biosynthesis “SULFATE.CYS.PWY..superpathway.of.sulfate.assimilation.and.cysteine.biosynthesis” was linked to increased risk for OA. Numerous studies have demonstrated that the gut microbiota exerts a direct or indirect influence on bone metabolism through mechanisms such as the gut–bone axis, involving the regulation of metabolic products, intestinal barrier function, immune responses, and endocrine activities.^[19,29–31]^ Dysbiosis of the gut microbiota has been closely associated with various bone metabolic disorders, including osteoporosis. Our identification of a causal link between the gut microbiota and OA further broadens the scope of research concerning the role of the gut microbiota in bone-related diseases, holding significant implications for future investigations in this field. Thus, this discovery showed new light on the complex pathophysiology of OA and offered potential insights for the development of innovative preventive and therapeutic strategies.

H_2_S, a molecule of significant importance, is synthesized by the gut microbiota and exerts a multitude of effects on human physiology.^[32]^ Certain bacterial species, including *Escherichia*, *Salmonella*, and members of the *Enterobacteriaceae* family, possess specialized enzymes that facilitate the production of H_2_S.^[33]^ Notably, gut bacterial are capable of generating H_2_S from a diverse range of substrates, with sulfate being a particularly favored source.^[34]^ The gut microbiota composition and its metabolic activities thus have a profound impact on endogenous H_2_S levels.^[35]^ Alterations in gut bacterial populations could lead to changes in H_2_S production and availability. Moreover, assimilatory sulfate reduction (ASR) is present in all living organisms and represents the process by which microorganisms utilize inorganic sulfate to synthesize organic sulfur compounds.^[36]^ During this process, sulfate is reduced to H_2_S and subsequently incorporated into the biosynthesis of cysteine and methionine.^[33]^ Certain bacteria such as *Escherichia coli* in the gut metabolize, metabolize sulfate into H_2_S through the ASR pathway, which is present in both bacteria and plants, and subsequently into sulfur-containing amino acids.^[37]^ Animal experiments have shown that intra-articular administration of H_2_S leads to a reduction in cartilage destruction and oxidative damage, supporting the use of slow-releasing H_2_S molecules as a supplementary treatment for OA.^[38]^ However, current studies have not yet included research for causal determination. Our research showed that the gut bacterial pathway abundance ‘SO4ASSIM.PWY..sulfate.reduction.I..assimilatory.’ was a protective factor for KOA. This finding provided a fresh perspective on causal inferences in OA. This result indicates that the proper functioning and abundance of this gut bacterial pathway may contribute to maintaining optimal H_2_S levels, which in turn could have a beneficial impact on OA development and progression.

In contrast, another gut bacterial pathway “SULFATE.CYS.PWY. superpathway.of.sulfate.assimilation.and.cysteine.biosynthesis” served as a risk factor for OA might not be beneficial to maintaining optimal H_2_S concentration, which could contribute to OA pathogenesis. Bacteria convert sulfate into H_2_S, which is subsequently utilized for the biosynthesis of cysteine and methionine, specifically through the superpathway of sulfate assimilation and cysteine biosynthesis, as well as the ASR pathway.^[33]^ Sulfate assimilation is a crucial pathway for microorganisms to acquire sulfur, and cysteine is a key amino acid in numerous biological processes.^[33,39]^ The biosynthesis of cysteine is contingent upon the synergistic action of O-acetylserine sulfhydrylase and serine acetyltransferase, 2 key enzymes that facilitate the incorporation of sulfur into the cysteine molecule.^[40]^ Furthermore, the cysteine synthesis pathway in bacteria is intimately linked to the host’s sulfate assimilation process, highlighting the metabolic interplay between microbes and their hosts.^[41]^ However, further studies are needed to elucidate the detailed molecular mechanisms underlying this relationship. Therefore, understanding the gut bacterial-H_2_S axis holds the potential to furnish a crucial foundation for deciphering the mechanisms underlying the progression of OA. By potentially targeting the gut microbiota to regulate H_2_S levels, it may subsequently mitigate the symptoms of joint disorder, thus opening up novel avenues for therapeutic interventions in the field of OA.

MR, by using genetic variants as IV, has emerged as a powerful approach in OA research.^[42]^ It allows for more reliable causal inference compared with traditional observational studies by minimizing confounding factors. This is crucial as we explore the roles of gut bacterial pathways and H_2_S in OA. Our study provides evidence that the gut bacterial pathway abundance associated with H_2_S “SO4ASSIM.PWY..sulfate.reduction.I.assimilatory.” acts as a protective factor for OA. The presence of pleiotropy may introduce bias, thereby undermining the accuracy of causal inference.^[43]^ To overcome this challenge, we employed various sensitivity analysis methods to verify the robustness of MR analysis. These methods include MR-PRESSO and MR-Egger intercept analysis, both of which did not detect the presence of pleiotropy, a common issue in MR studies.^[44]^ Furthermore, the results of Cochran *Q* test and symmetric funnel plots also indicated that our study lacks both pleiotropy and heterogeneity, further confirming that our IVs were valid and reliable indicators of H_2_S exposure. The directional analysis from the scatter plots of the 5 MR algorithms further supported the consistency of our results. However, it should be noted that the majority of MR analyses have been conducted in European populations, which restricts the generalizability of our research findings, and the interaction between H_2_S and the gut microbiota in relation to OA is highly complex and still requires further validation through in vivo and in vitro experiments. Understanding the causal links can enhance our knowledge of OA etiology and provide a theoretical basis for targeted preventive and therapeutic interventions.

## 5. Conclusion

This study investigated the potential causal relationship between H_2_S-related exposure factors and OA using MR analysis. By examining GWAS data from the IEU OpenGWAS project, we identified that specific intestinal bacterial pathways involved in sulfate reduction may act as either protective or risk factors for the development of OA. These findings not only confirm the association between H_2_S and OA but also suggest a potential causal link between certain gut microbiota metabolic pathways and the onset of the disease. This insight offers a novel therapeutic approach for managing OA through modulation of the gut microbiota. Targeted regulation of sulfate-reducing intestinal bacteria and their associated metabolic activities may help prevent or alleviate OA symptoms, particularly in patients diagnosed with KOA. Future studies should validate these observed causal relationships through clinical trials and explore strategies to slow or prevent OA progression by modifying the composition or function of the gut microbiome. Clinical research could assess the applicability of hydrogen sulfide and its related microbial pathways in OA patients potentially establishing them as innovative treatment modalities. In summary, this study contributes new evidence to the understanding of how hydrogen sulfide and gut microbiota influence osteoarthritis. Further investigation into the underlying mechanisms and their clinical translation is warranted to facilitate the development of novel therapeutic interventions.

## Acknowledgments

We appreciate all investigators for providing publicly summary data.

## Author contributions

**Conceptualization:** Peng Yang, Jun Ma, Jiuyi Sun.

**Data curation:** Peng Yang, Jun Ma.

**Formal analysis:** Ye Jiang, Xiaofei Ye.

**Funding acquisition:** Ye Jiang.

**Investigation:** Xiaofei Ye.

**Methodology:** Peng Yang, Xiaofei Ye, Lina Liu.

**Resources:** Xiaofei Ye, Lina Liu, Jiuyi Sun.

**Software:** Ye Jiang, Xiaofei Ye.

**Supervision:** Ye Jiang, Xiaofei Ye.

**Visualization:** Jun Ma, Jiuyi Sun.

**Writing – original draft:** Peng Yang, Ye Jiang.

**Writing – review & editing:** Jun Ma, Jiuyi Sun.

## Supplementary Material


